# Crosstalk Between Inflammatory Signaling and Methylation in Cancer

**DOI:** 10.3389/fcell.2021.756458

**Published:** 2021-11-24

**Authors:** Dipanwita Das, Nandini Karthik, Reshma Taneja

**Affiliations:** Department of Physiology, Healthy Longevity Translational Research Program, Yong Loo Lin School of Medicine, National University of Singapore, Singapore, Singapore

**Keywords:** cancer, inflammation, epigenetics, DNA methylation, histone methylation 2

## Abstract

Inflammation is an intricate immune response against infection and tissue damage. While the initial immune response is important for preventing tumorigenesis, chronic inflammation is implicated in cancer pathogenesis. It has been linked to various stages of tumor development including transformation, proliferation, angiogenesis, and metastasis. Immune cells, through the production of inflammatory mediators such as cytokines, chemokines, transforming growth factors, and adhesion molecules contribute to the survival, growth, and progression of the tumor in its microenvironment. The aberrant expression and secretion of pro-inflammatory and growth factors by the tumor cells result in the recruitment of immune cells, thus creating a mutual crosstalk. The reciprocal signaling between the tumor cells and the immune cells creates and maintains a successful tumor niche. Many inflammatory factors are regulated by epigenetic mechanisms including DNA methylation and histone modifications. In particular, DNA and histone methylation are crucial forms of transcriptional regulation and aberrant methylation has been associated with deregulated gene expression in oncogenesis. Such deregulations have been reported in both solid tumors and hematological malignancies. With technological advancements to study genome-wide epigenetic landscapes, it is now possible to identify molecular mechanisms underlying altered inflammatory profiles in cancer. In this review, we discuss the role of DNA and histone methylation in regulation of inflammatory pathways in human cancers and review the merits and challenges of targeting inflammatory mediators as well as epigenetic regulators in cancer.

## Introduction

### Inflammation

The immune system protects the human body from different infections and can respond to cellular damage. Chiefly, the immune system plays a central role in clearing infection, healing an injury, and restoring tissue homeostasis. Inflammation is a complex immune defense response triggered to neutralize an invading infection and is characterized by redness, swelling, and pain (Coussens and Werb, 2002). Inflammation is mediated and regulated by different cytokines. Pro-inflammatory and anti-inflammatory cytokines function in an opposing manner, the former triggering the inflammatory reaction whereas the latter reduces the response. The fate of the cell depends on the balance between the pro- and anti-inflammatory immune signals. Acute inflammatory response is beneficial to the host and a well-balanced immune response can be largely anti-tumorigenic (Yasmin et al., 2015). Chronic activation of inflammatory response is, however, linked to pro-tumorigenic conditions and cancer. Inflammation and cancer are closely linked. Individuals with chronic inflammatory diseases have a higher risk of developing cancer (Nelson et al., 2004; Garcea et al., 2005; Vagefi and Longo, 2005; Peek and Crabtree, 2006). Studies suggest that around 20% of cancers are associated with chronic inflammation that is linked to different stages of oncogenesis: cellular transformation, tumor progression, invasion, angiogenesis, and metastasis (Coussens and Werb, 2002; Mantovani, 2005; Vendramini-Costa and Carvalho, 2012).

The process by which a normal cell is transformed into a pre-malignant cell is known as tumor initiation. The proliferation of genetically altered cells and chronic inflammation promotes tumor growth by inhibiting apoptosis and accelerating angiogenesis. Tumor progression and metastasis, which involves additional genetic changes, increased tumor size, and spreading of the tumor from the local site to different secondary sites, is influenced by inflammation. Thus, there is a close link and a continuous crosstalk between inflammation and cancer at all stages of tumorigenesis (Grivennikov et al., 2010).

Genetic and epigenetic alterations trigger transformation of normal cells to cancer cells (Baylin and Jones, 2016). Inflammatory signaling pathways that get activated in different cancers is an important connecting link between chronic inflammation and oncogenesis. The molecular circuits that lead to sustained activation of inflammatory factors are still being explored. Several epidemiological and molecular studies link cancer and inflammation. Proinflammatory cytokines including chemokines and adhesion molecules cause chronic inflammation. Proinflammatory genes like Tumor necrosis factors (TNFs) and members of its superfamily, members of interleukin family, vascular endothelial growth factor (VEGF), matrix metalloprotease 9 (MMP-9), 5-lysyl oxidase (5-LOX), and cyclooxygenase-2 (COX-2) are important players of apoptosis, angiogenesis, proliferation, invasion, and metastasis (Yasmin et al., 2015). Many signaling pathways, including IL-6/STAT3 (interleukin-6/signal transducer and activator of transcription 3), play crucial roles in cancer initiation and progression. Inflammatory cytokines like IL-6 and interferons (IFNs) activate STAT3 and induce its translocation to the nucleus, where it binds to specific regulatory sites to activate gene expression. In oncogenic conditions, STAT3 is constitutively activated leading to sustained expression of its downstream targets, which are involved in cell proliferation, invasion, and differentiation (Yu et al., 2009, p. 3). Thus, persistent activation of inflammatory mediators can cause tumor progression and may be triggered by events of aberrant epigenetic changes.

Epigenetic alterations are essential hallmarks to cancer initiation and progression. However, what triggers epigenetic changes in cancer is still being investigated. Mechanistic insights of regulation of inflammatory signaling by epigenetic alteration need an in-depth exploration to design effective therapeutic targets for different cancers. These targets include various mediators of inflammatory networks.

### Markers and Mediators of Inflammation

**FIGURE 1:**
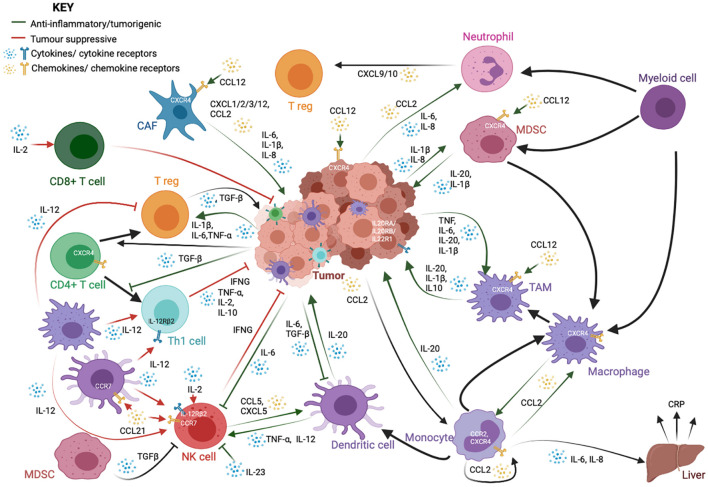
A schematic showing the complex interactions between cancer cells and immune cells, particularly those in the tumor microenvironment. While immune cells, such as cytotoxic CD8^+^ T-cells, T helper (Th1) and natural killer (NK) cells, are important for anti-tumor responses and cancer cell clearance, tumors and cancer-associated fibroblasts (CAFs) often employ a variety of signaling molecules that result in the dysregulation of a variety of both lymphoid and myeloid-derived immune cells. The crosstalk between these cells further dampens the anti-tumor response and exacerbates oncogenic phenotypes such as cancer cell proliferation and metastasis. This signaling network is reliant upon a whole host of cytokine and chemokine ligands and their receptors. These cytokines and chemokines could also be present in the tumor microenvironment as a result of chronic inflammation, and release from the tumor and infiltrating tumor cells could lead to persistent inflammation in turn. The factors shown here are only a part of the many factors involved in cell-to-cell crosstalk. They have been highlighted here because their expression is regulated by methylation.

The inflammatory response to infection or injury comprises a whole host of immune cells, secreted factors, signaling pathways, and markers. It is important to identify the major markers, mediators, and orchestrators of this intricate network, and how they relate to each other in order to appreciate the nature and complexity of the epigenetic control of the network (Figure 1).

#### Tumor Necrosis Factor Alpha

Tumor necrosis factor alpha (TNFα) is a member of the tumor necrosis factor (TNF) family and a major signaling molecule involved in the inflammatory network. It is a potent cytokine involved in the acute phase of inflammation that can trigger a cascade of signaling, resulting in the production of adhesion molecules that cause migration of neutrophils to the site of infection. TNFα is produced primarily by macrophages and has chemotactic roles. It signals through two transmembrane receptors, TNFR1 and TNFR2, and plays a key role in cell survival, proliferation, and apoptosis. As a master regulator in the cytokine cascade, TNFα levels are under tight control. This regulation is context- and tissue-specific, but several epigenetic mechanisms have been identified to be critical. TNFα is found to be aberrantly expressed in many diseases, including autoimmune diseases and cancer (Parameswaran and Patial, 2010; Chu, 2013). It has been shown to have both tumor-suppressive and tumor-promoting roles (Waters et al., 2013; Montfort et al., 2019).

#### Interleukins

Interleukins (ILs) (from IL-1 to IL-38) are cytokines produced during inflammatory processes, primarily by macrophages and monocytes at the site of inflammation. They drive the production of acute phase proteins linked to inflammation. ILs are also well-known to be deregulated in a whole range of inflammation-linked pathologies, including cancer (Gabay, 2006; Akdis et al., 2016). In cancer, IL-20, which is typically secreted by monocytes, macrophages, and dendritic cells, promotes pro-inflammatory signaling, metastasis, and proliferation. The IL20 family receptors are found to be expressed on a variety of cancer cell lines (Rutz et al., 2014; Niess et al., 2018; Figure 1). IL-1β is likewise produced by both tumor cells as well as infiltrating monocyte-derived suppressor cells (MDSCs) and tumor-associated macrophages (TAMs), thereby recruiting and activating other myeloid cells and regulatory T cells (T regs), as well as promoting tumor cell proliferation and angiogenesis (Bent et al., 2018). IL1β and other pro-inflammatory cytokines, such as IL-6 and IL-8, which are known to promote inflammatory signaling, tumor growth, and metastasis, are also produced by cancer-associated fibroblasts (CAFs) (Tanaka et al., 2014; David et al., 2016; Bent et al., 2018). IL-6 also modulates the activity of macrophages, T regs, natural killer (NK) cells, and antigen-presenting dendritic cells (DCs) (Jones and Jenkins, 2018). IL-12, produced mainly by macrophages and DCs, is thought to be tumor suppressive through its ability to stimulate Interferon-γ (IFNγ) production by Th1 cells and NK cells. IL-2 is likewise primarily tumor suppressive in nature, through its activation of T cells and NK cells (Choudhry et al., 2018). IL-23 is overexpressed in several cancer models and promotes tumorigenesis through suppression of NK cell activity, activation of IL-17 signaling, and the upregulation of MMP9 and VEGF (Ngiow et al., 2013; Yan et al., 2018; Figure 1).

#### Chemokines

Chemokines constitute a family of secreted chemotactic proteins, including 50 known endogenous ligands and 20 known receptors that signal through cell surface G protein-coupled chemokine receptors and can stimulate the migration of cells, especially leukocytes. Chemokines can be secreted by a wide variety of cells and play a pivotal role in the development of the immune system as well as in inflammatory responses (Griffith et al., 2014; Hughes and Nibbs, 2018). Pro-inflammatory cytokines, such as CXCL1/2/3/12 and CCL2 are produced by CAFs in the tumor microenvironment (Bent et al., 2018). Neutrophils can produce CXCL9 and CXCL10 to recruit T-cells to the microenvironment (David et al., 2016). The CXCL12/CXCR4 axis is important for tumor cell survival and metastasis, CAF activation, and recruitment of monocyte-derived cells (Chatterjee et al., 2014) CCL21 signaling; on the other hand, it can recruit lymphocytes, NK cells, and antigen presenting cells with anti-tumor activity (Lin et al., 2014; Figure 1).

#### Interferon-γ

Inflammation is a critical part of the immune response to harmful pathogens. Therefore, paradoxically, although chronic inflammation may play a role in tumorigenesis and tumor progression, impaired acute inflammation in response to viruses may in fact be instrumental in tumor development. One example of an inflammatory molecule critical for prevention of oncogenic signaling is IFNγ, which is the principal macrophage-activating cytokine produced by a whole host of immune cells in response to foreign antigens, particularly viruses. IFNγ is required for the expression of human major histocompatibility complex (MHC) class I and class II proteins, and therefore, plays a critical role in tumor immunogenicity. Unsurprisingly therefore, its expression is reduced in various forms of cancer (Schroder et al., 2004; Castro et al., 2018).

#### Transforming Growth Factor Beta

Transforming growth factor beta (TGF-β) is a key pleiotropic cytokine involved in many pathways, including inflammation and immune response. It regulates lymphocyte proliferation, differentiation, and survival and also controls inflammatory responses through the regulation of chemotaxis, activation, and survival of a variety of immune cells, including lymphocytes, natural killer cells, dendritic cells, macrophages, mast cells, and granulocytes (Li et al., 2006). In cancer, TGF-β signaling typically provides a favorable microenvironment for tumor growth through regulation of infiltration of inflammatory cells and cancer associated fibroblasts (Yang et al., 2010). It promotes expansion of T regs, inhibits expansion and/or activity of effector T cells, DCs, and NK cells and regulates macrophages and neutrophils (Batlle and Massagué, 2019; Figure 1). It can induce the expression of DNMTs and, therefore, can significantly affect the global methylome of cancer cells.

#### Signal Transducers and Activators of Transcription

Signal transducers and activators of transcription (STATs) are transcription factors involved in many signaling networks that often involve ILs and Janus kinases (JAKs). They are critical components of the response to infection. Activated by interferon signaling, STATs promote inflammation in a myriad of ways, including through the induction of chemokine expression, reactive oxygen species and NO, and the regulation of the development and death of hematopoietic cells (Pfitzner et al., 2004; Kaplan, 2013).

#### Nuclear Factor Kappa-Light-Chain-Enhancer of Activated B Cells

Nuclear factor kappa-light-chain-enhancer of activated B cells (NF-κB) is a major transcription factor at the nexus of inflammatory signaling and cancer. NF-κB is a critical regulator of both adaptive and innate immunity through its ability to induce the secretion of pro-inflammatory genes and regulation of the inflammasome. Its downstream targets include TNFα and IL6 (Liu et al., 2017; Xia et al., 2018). NF-κB is often deregulated in cancers.

#### Cyclooxygenase-2

Cyclooxygenase-2 is one of the two closely related enzymes responsible for converting arachidonic acid to prostaglandins, including prostaglandin E2 (PGE2). COX-2 is induced in response to inflammatory stimuli and is involved in the production of those PGEs that mediate pain and support inflammation (Simon, 1999). COX-2 derived PGE2 has been linked to various stages of the process of tumorigenesis and progression. COX-2 can be induced by pro-inflammatory TNF-α, IL-1, and IFN-γ, and suppresses anti-inflammatory IL-4, IL-13, and IL-10 (Harizi, 2015).

#### C-Reactive Protein

C-reactive protein (CRP) is a protein produced in acute response to inflammation. It is a sensitive marker of chronic low-grade inflammation that can be detected in the blood. CRP also plays key roles in apoptosis, phagocytosis, the complement pathway, production of nitric (NO), and the production of interleukin-6 and tumor necrosis factor-α (Sproston and Ashworth, 2018).

#### Suppressor of Cytokine Signaling 1

Suppressor of cytokine signaling (SOCS) proteins are suppressors of cytokine signaling, particularly through the JAK/STAT pathway, and negative regulators of inflammatory responses (Duncan et al., 2017; Liau et al., 2018).

### Epigenetics

Activating inflammatory signaling cascades in response to cues need to be tightly regulated. Epigenetic mechanisms play a critical role in regulating inflammatory signaling pathways. They provide a means by which the expression of genes in specific pathways can be turned on or off reversibly and in a controlled manner.

Epigenetic mechanisms can take multiple forms. Prominent among these are DNA and histone modifications. Both these forms of regulation affect the interactions between DNA and the nucleosomes. Tight DNA-histone interactions prevent binding of transcriptional machinery, whereas more relaxed interactions lead to increased accessibility and facilitate active transcription. Through DNA and histone modifications, these interactions, and consequently, gene transcription, can be regulated at specific loci.

#### DNA Methylation

CpG islands are dinucleotide repeats that are abundantly present in mammalian genomes and are associated, in the unmethylated form, with gene promoters. DNA methylation involves the conversion of cytosine residues in DNA to 5-methylcytosine (5mC) by the transfer of a methyl group from the cofactor S-adenyl methionine (SAM). This process is carried out by a family of enzymes known as DNA methyltransferases (DNMTs) comprising DNMT1, DNMT2, DNMT3a, DNMT3b, and DNMT3L. 5mC is a major repressive mark, because it prevents DNA transcription either through the recruitment of repressive complexes or through prevention of transcription factor binding. Therefore, DNA methylation is a critical form of transcriptional silencing in many physiological and developmental processes. The opposite function, that is, DNA demethylation, is carried out by a group of enzymes known as tet-eleven translocation (TET) proteins. These proteins catalyze the conversion of 5mC to 5mC into 5-hydroxymethylcytosine (5hmC). This is further oxidized to form 5-formylcytosine (5fC) and 5-carboxylcytosine (5caC), which can then be converted back to unmodified cytosine (Tahiliani et al., 2009; He et al., 2011; Ito et al., 2011). The opposing functions of DNMTs and demethylases are necessary for many processes including heterochromatin maintenance, tissue-specific gene expression, genomic imprinting, X-chromosome inactivation and transcriptional silencing of retroviral elements. Deregulation of the expression or function of either of these groups of enzymes can have widespread consequences on cells (Jin et al., 2011; Jones, 2012; Greenberg and Bourc’his, 2019). Global and gene-specific hypomethylation, as well as regional hypermethylation, have been implicated in cancer (Ehrlich, 2002; Szyf, 2003).

#### Histone Methylation

Like DNA methylation, histone methylation is also a crucial regulatory process. Histone residues, particularly lysine (K) and arginine (R) on histone subunit 3 (H3), can acquire a large variety of post-translational modifications (PTMs) that can affect the structure of the subunit and the function of the nucleosome as a whole. Among these PTMs, which include methylation, acetylation, formylation, propionylation, butyrylation, crotonylation, malonylation, succinylation, hydroxylation, ubiquitination, sumoylation, adenosine diphosphate (ADP)-ribosylation, citrullination, and glycosylation, histone methylation/demethylation is perhaps the most well studied.

Lysine and arginine residues can accept between 1 and 3 methyl (me) groups, added sequentially, from SAM. The effect of histone methylation depends on the number of groups added and the residue modified. Some marks, such as H3K9me1/2 and H3K27me3, are repressive in nature, whereas others, such as H3K4me2, facilitate transcriptional activation. Enzymes that catalyze the addition of methyl groups are known as lysine methyltransferases (KMTs), whereas those that have the opposite function are known as demethylases. Specific enzymes catalyze the formation or removal of only specific marks. Likewise, epigenetic “readers” that recognize these marks and relay the effects to transcriptional complexes are also specific in their recognition of marks. Therefore, the entire process is a tightly regulated and involves many different enzymes (Bannister and Kouzarides, 2011; Greer and Shi, 2012; Zhao and Shilatifard, 2019).

Due to the importance of DNA and methylation as forms of regulation of gene expression, the deregulation of the enzymes involved in these processes has profound effects on the physiology of a cell, including in the context of cancer. Deregulated expression of many epigenetic modifiers, including methyltransferases and demethylases, have been linked to perturbed gene expression profiles, oncogenic phenotypes, and poor survival outcomes in cancer patients. Histone demethylases are capable of removing methyl groups from both histones and proteins. KDM1 family of demethylases is composed of KDM1A and KDM1B. KDM1A also known as LSD1 (Lysine-specific demethylase 1) (Shi et al., 2004) removes methyl groups via amine oxidase domain activity using FAD cofactor. The second group of histone demethylases is the Jumonji C (JmJC) domain containing demethylases that remove trimethylation mark (D’Oto et al., 2016). Cancer cells can also hijack the activity of these enzymes to alter the expression of genes involved in inflammatory signaling cascades.

In this article, we review the regulation of inflammatory signaling through both histone and DNA methylation in cancer. We discuss the various signaling cascades that cancer cells employ, through the use of altered histone and DNA methylation, to adopt an inflammatory phenotype that allows survival, colonization, and metastasis. It is important to note that the reverse also occurs; epigenetic profiles can change in response to inflammation. We also discuss targeting inflammation using small molecule inhibitors of various key players as an alternative to direct targeting of components of signaling pathways. Finally, we highlight recent progress, future challenges, and what we can learn from other diseases that can help with development of therapeutics in cancer.

## Dna and Histone Methylation in Regulation of Inflammatory Signaling Pathways

Global reorganization of epigenetic modifications is an important part of cancer initiation and progression, including in the switching on of pro-inflammatory signaling programs in cancers cells and infiltrating tumor cells.

As a major repressive mark and regulator of gene expression, DNA methylation is of critical importance in switching on and off inflammatory signaling pathways in response to cues. Several studies have indicated interactions between DNA methylation and circulating inflammatory proteins (Ahsan et al., 2017; Myte et al., 2019). Epigenome wide association studies (EWAS), global DNA methylation patterns, and candidate gene analysis suggested that global genome hypomethylation is linked to inflammation (Gonzalez-Jaramillo et al., 2019). Several groups have studied the association between global DNA methylation (LINE-1 methylation) patterns and CRP levels. Some have reported no association between global DNA methylation and CRP levels (Baccarelli et al., 2010; Zhang et al., 2012), while others have indicated a link between lower methylation and higher CRP levels (Perng et al., 2012). In addition, meta-analysis of several EWAS has shown that many specific differentially methylated CpG islands are significantly linked to CRP expression and chronic low-grade inflammation. Serum levels of CRP were linked either positively or negatively to various CpG sites, notably with one site near the transcription start site of the Absent in melanoma 2 (AIM2 gene), a protein induced by interferon-gamma and involved in the innate immune response (Ligthart et al., 2016). There appears to be a link between higher CRP levels and lower levels of AIM2 and IL6 methylation, as well as between higher CRP levels and higher levels of suppressor of cytokine signaling 1 (SOCS1), LY86 and EEF2 methylation [reviewed by Gonzalez-Jaramillo et al. (2019)].

Histone methylation is also important in propagating inflammatory cues. H3K9 methyl transferases and demethylases balance the methylation status of H3K9. Jmjd3, a Jumonji family member, is responsible for the deletion of histone marks and control of differentiation and cell identity in macrophages. Thus, Jmjd3 protein functions as a link between inflammation and reprogramming of the epigenome (Ishii et al., 2009). Macrophages exposed to bacterial products and inflammatory cytokines induce Jmdj3, which in turn binds to polycomb group (PcG) target genes and regulates their H3K27me3 levels and the transcriptional activity (De Santa et al., 2007). Activation of Jmjd3 on continuous IL-4 treatment triggers release of H3K27me3 repressive marks from STAT6 promoter. Jmjd3 is positively regulated by activated STAT6 through promoter binding. Jmjd3 also triggers expression of specific inflammatory genes by removal of H3K27me mark (Bayarsaihan, 2011).

**FIGURE 2:**
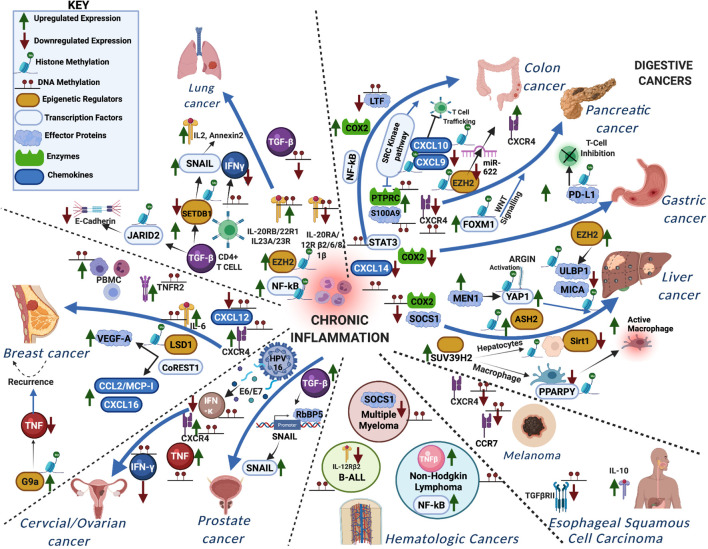
A schematic representation showing how aberrant DNA and histone methylation affect the different inflammatory signaling pathways in various forms of cancer. The different proteins functioning in the chronic inflammatory network are either methylated or trigger methylation of other proteins leading to the different cancers.

DNA and histone methylation defects affect inflammatory signaling in a myriad of ways in various forms of cancer. Cooperative interactions between DNA methylation and histone methylation during severe systematic inflammation (SSI) was shown in TNFα promoter in blood leucocytes (Gazzar et al., 2008). Here, we summarize the known changes to these pathways in individual forms of cancer, to show the many ways in which they are deregulated and underline the need to take into account the various factors involved in inflammation and tumor progression when trying to treat certain forms of cancer (Table 1 and Figure 2).

**TABLE 1:**
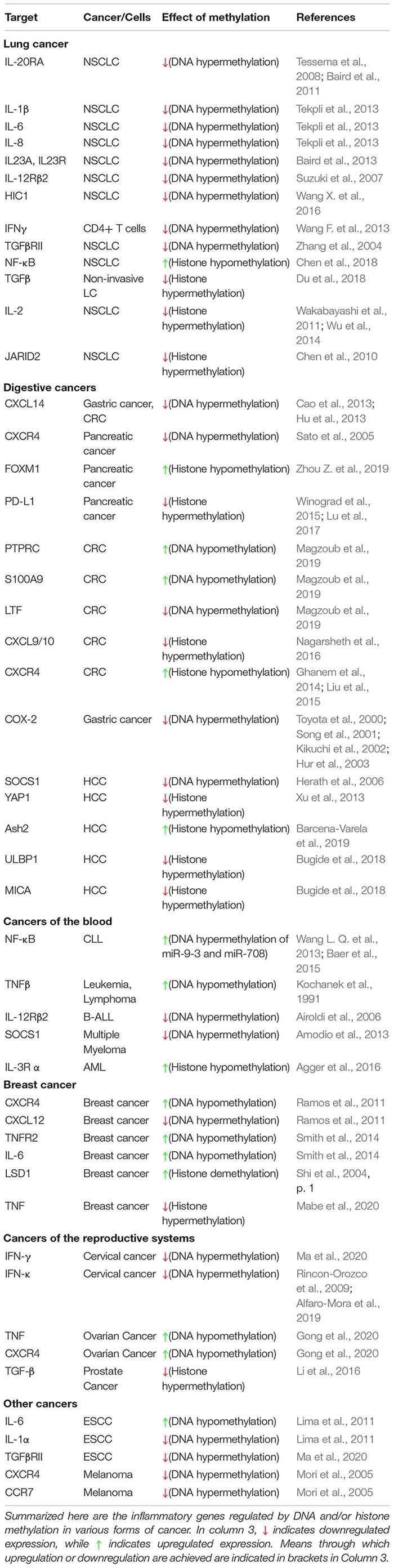
DNA and histone methylation in regulation of inflammatory signaling pathways.

### Lung Cancer

Lung cancer is one of the most common and lethal cancers. Small-cell lung cancer accounts for about 10–15% of global incidence, while non-small-cell lung cancers (NSCLCs) account for the majority, with subsets of patients showing mutations in various genes, including Epidermal growth factor receptor (EGFR), Anaplastic lymphoma kinase (ALK), ROS1, and Neurotrophin receptor kinase (NTRK). Despite considerable progress in developing biomarkers and stratifications for treatment strategies, NSCLC continues to be a leading cause of death worldwide (Cersosimo, 2002; Duma et al., 2019).

#### DNA Methylation

The expression of IL-20 and its receptors are often found to be dysregulated in NSCLC. IL20 can signal through IL-20RA/RB or IL20-RB/IL22-R1 receptor complexes. These genes are epigenetically regulated through several mechanisms, including DNA methylation. IL-20RB and IL-22R1 were found to be overexpressed at both the mRNA and protein levels when compared to healthy counterparts. Conversely, loss of IL-20RA expression, linked to promoter hypermethylation, was found when compared to normal bronchial epithelial cells. This may suggest that the IL20-RB/IL22-R1 complex is the main complex through which NSCLC cells signal. IL-20RA has been previously linked to anti-angiogenic effects in NSCLC, and restoration of IL20 signaling through IL20RA was shown to downregulate VEGF expression (Tessema et al., 2008; Baird et al., 2011). A study by Tekpli et al., has shown that pro-inflammatory IL-1β, IL-6, and IL-8 genes all show differential DNA methylation patterns in NSCLC when compared to adjacent non-cancerous tissue or bronchial epithelial cells, and there is an inverse relationship between DNA methylation and gene transcription for IL6 and IL1β. Interestingly, all these cytokines were found to be hypermethylated and downregulated in tumor tissues when compared to non-tumor tissues (Tekpli et al., 2013). IL23A, a member of the IL6 family of cytokines, is pro-proliferative in NSCLC, and treatment with 5-aza-2’deoxycitidine (5-Aza) showed an increase in the expression of IL23A, indicating that it is transcriptionally silenced through DNA methylation. Similarly, the expression of the IL23 receptor (IL23R) was also increased upon 5-Aza treatment in the A549 lung adenocarcinoma cell line (Baird et al., 2013). In lung adenocarcinomas, aberrant methylation of the IL-12Rβ2 gene was linked to loss of expression in cell lines and primary tumors and poor prognosis among adenocarcinoma patients. Treatment with a demethylating agent was able to restore expression in these cell lines (Suzuki et al., 2007). In addition, the hypermethylation and loss of Hypermethylated in cancer 1 (HIC1) was shown to lie upstream of upregulation of IL6 and activation of the IL6/STAT3 axis (Wang X. et al., 2016).

Co-culturing SPC-A1 lung cancer cells and healthy CD4+ T cells induced DNMT expression and IFNγ promoter hypermethylation in CD4+ T cells, indicating a tumor-induced, DNA methylation-dependent suppression of IFNγ in lung cancer and highlighting the crosstalk between these processes (Wang F. et al., 2013).

TGFβRII, which is thought to function as a tumor suppressor in many solid tumors, where TGF-β signaling is important for inhibition of epithelial cell growth, is downregulated in NSCLC through methylation of its promoter (Zhang et al., 2004).

#### Histone Methylation

A number of NSCLCs show high levels of Enhancer of Zeste 2 (EZH2), the enzymatic subunit of polycomb repressive complex 2 (PRC2). Tumors from mice administered with EZH2 inhibitors singly or in conjunction with chemotherapy were sensitive to EZH2 inhibition. Along with it, there was an amplification of an inflammatory program involving NF-KB signaling. Combinatorial therapy of EZH2 inhibitors with anti-inflammatory agents can provide a promising therapy for a subset of Kras-driven NSCLC (Chen et al., 2018).

In non-invasive lung tumor cells, TGF-β facilitates the association of methyltransferase SET Domain Bifurcated Histone Lysine Methyltransferase 1 (SETDB1) with Smad3 which in turn mediates H3K9me in Snail promoter, downregulating its expression. In invasive lung tumor cells undergoing TGF-β-induced EMT, Snail promoter is de-repressed due to the repression of SETDB1 (Du et al., 2018). The interaction of SETDB1 with Smad3 reduces metastasis in lung cancer by downregulating IL-2 and the calcium-dependent RNA-binding protein annexin A2 (ANXA2), which interacts with c-myc mRNA (Wakabayashi et al., 2011; Wu et al., 2014).

Jumonji and At-rich interaction domain containing 2 (JARID2), a component of the PRC2 complex is activated by TGF-β and downregulates expression of E-cadherin in lung cancer cells. JARID2 occupies promoters of CDH1 and miR-200 family members which in turn controls the recruitment of PRC and G9a methyltransferase, promoting methylation of H3K27 and H3K9 (Li et al., 2016). The pro-metastatic effect of G9a is contributed by gene silencing of epithelial cell adhesion molecules (ep-CAM), increasing the invasive capacity of lung tumor cells (Chen et al., 2010).

### Digestive Cancers

Digestive cancers, including liver cancer, gastric cancer, pancreatic cancer, and colon/colorectal cancers, are a leading cause of cancer-related deaths worldwide. Hepatocellular carcinoma (HCC) is one of the most common cancers and is chiefly caused by infectious diseases like viral hepatitis B or C or through food toxins. In the early stages, inflammatory responses like cytokine secretion or proliferation play important roles in subsequent development of HCC. Chronic inflammatory responses like liver cirrhosis and necrosis are important in the later stages and advancement of HCC. Epigenetic regulation is important in both the early and late stages of HCC.

#### DNA Methylation

The chemokine CXCL14 is downregulated in gastric cancer cells when compared to healthy tissue, due at least in part to aberrant hypermethylation in exon 1 of the gene (Hu et al., 2013), and in colorectal cancer, where hypermethylation and loss of CXCL14 expression are linked to proliferation, migration, invasion, and EMT through NF-κB (Cao et al., 2013). The chemokine receptor CXCR4 is hypermethylated in pancreatic cancer cell lines and primary pancreatic adenocarcinomas, while it is unmethylated in healthy pancreas tissue. The reason for CXCR4 downregulation in pancreatic cancer is unclear. It has been suggested that the *de novo* methylation of the CXCR4 locus may simply be a part of genome-wide process in a distinct subgroup of pancreatic cancers characterized by a profound methylator phenotype and that alternative pathways to CXCR4/CXCL12 may be utilized for tumor progression (Sato et al., 2005).

Nuclear factor kappa-light-chain-enhancer of activated B cells and STAT3 are both critical in the progression of chronic inflammation to malignancy in CRC, primarily through the maintenance of a favorable microenvironment for tumorigenesis through secretion of a myriad of pro-inflammatory cytokines (Yang et al., 2019).

In addition, the Protein Tyrosine Phosphatase Receptor Type C (PTPRC) gene, which disrupts normal T- and B-cell signaling through SRC kinase pathways, and the S100 Calcium Binding Protein A9 (S100A9) gene, which is implicated in many conditions associated with inflammation, are both hypomethylated. At the same time, the promoter of the LTF (Lactotransferrin) gene, which restricts the inflammatory reaction in CRC, is hypermethylated (Magzoub et al., 2019).

While some studies have linked high COX-2 expression to worse outcomes in CRC (Soumaoro et al., 2004; Ogino et al., 2008; Wang and Dubois, 2010), there is a subset of gastric cancers and CRCs where the expression of COX-2 is lost through hypermethylation of the promoter (Toyota et al., 2000; Song et al., 2001; Kikuchi et al., 2002; Hur et al., 2003). Consequently, treatment with 5-Aza restores COX-2 expression and sensitivity to IL1β signaling (Song et al., 2001). Interestingly, in HCC, downregulation of COX-2 was linked to reduced survival of patients (Fernández-Alvarez et al., 2012).

Furthermore, hypermethylation and loss of SOCS1 expression are a common occurrence in HCC (Herath et al., 2006).

#### Histone Methylation

Higher levels of H3K4 trimethylation are associated with a poor prognosis of HCC (Chen et al., 2020). Menin is a scaffold protein encoded by multiple endocrine neoplasia type1 (MEN1) gene. MEN1 undergoes heterozygous ablation in female mice and causes a reduction in chemical carcinogen-induced liver carcinogenesis and suppresses the activation of inflammatory pathways. Chromatin immunoprecipitation assays revealed that menin and H3K4me3 occupancy at the YAP1 promoter was markedly increased in HCC tissues (Xu et al., 2013). Abundantly expressed proteoglycan, Argin in the HCC tissue can activate the YAP gene and cause metastasis and invasion. On the other hand, low levels of H3K4 dimethylation in HCC are associated with Ash2, an H3K4 methylating enzyme (Barcena-Varela et al., 2019). A study showed that EZH2 overexpression is associated directly with promoters of natural killer (NK) cells ligand like ULBP1 and MICA in HCC cells and promotes the occupancy of H3K27me3 repressive marks in these promoters (Bugide et al., 2018).

Suppressor of variegation 3–9 homolog 2 (SUV39H2) or KMT1B-mediated H3K9me3 accelerates hepatocarcinogenesis by contributing to non-alcoholic steatohepatitis in mice. KMT1B represses Sirt1 transcription in hepatocytes, whereas KMT1B suppresses PPARΥ in macrophages which favors proinflammatory active macrophage (M1) phenotype over anti-inflammatory alternatively active macrophage (M2) phenotype, thus elevating hepatic inflammation (Fan et al., 2017). HBV X protein (HBx) decreases levels of H3K27me3 silence modification while increasing levels of activating histone modification H3K27me1 in the host EpCAM promoter, which is involved in HBV-mediated hepatocarcinogenesis (Zhang et al., 2015, 2016).

In colon cancer, production of the Th1-type chemokines, CXC chemokine ligand 9 (CXCL9), and CXCL10, which mediates T cell trafficking, is inhibited by H3K27me3 in their gene promoters (Nagarsheth et al., 2016). However, CXC chemokine receptor 4 (CXCR4) is upregulated by EZH2-mediated loss of miR-622, thus favoring evasion of immune surveillance by interaction with CXCL12 (Ghanem et al., 2014; Liu et al., 2015).

Upregulation of FOXM1 by KMT4-induced H3K79me2 significantly reduces antitumor responses like bone marrow-derived dendritic cell (BMDC) maturation, T cell activation, and cytokine secretion via the Wnt5a signaling pathway in pancreatic cancer (Zhou Z. et al., 2019). Further expression of PD-L1, a T-cell inhibitory receptor ligand causing immunosuppression, was upregulated in pancreatic cancer due to upregulation of H3K27me3 levels in CD274 promoter triggered by KMT2A overexpression (Winograd et al., 2015; Lu et al., 2017).

### Cancers of the Blood

Cancers of the blood, the most common of which include leukemia, lymphoma, and myeloma, have seen a gradual increase in survival rates over time. Nevertheless, these cancers can still present various difficulties in treatment, and chemotherapy can lead to devastating long-term side effects, as well as relapse, in patients. Pro-inflammatory signaling molecules in the blood can affect a wide range of cells and trigger an intricate network of signaling, especially as hematopoiesis and leukocyte functions are dependent on cytokines and chemokines (Rosu-Myles et al., 2000; Allart-Vorelli et al., 2015).

#### DNA Methylation

In contrast to normal human granulocytes, monocytes, or HeLa cells, the TNFβ gene showed hypomethylation chronic myeloid leukemia (Kochanek et al., 1991).

In pediatric B-cell acute lymphoblastic leukemia (B-ALL) cells, methylation of a CpG island in exon 1 in the IL-12Rβ2 gene was found to be responsible for silencing of this gene. IL12 is an especially important anti-inflammatory, anti-tumorigenic signaling molecule that has been linked to restricting tumor growth through its anti-proliferative, anti-metastatic, and anti-angiogenic functions in various types of cancers (Dias et al., 1998; Duda et al., 2000; Pistoia et al., 2009). Therefore, inhibition of IL12 signaling through the ablation of one of its receptor subunits gives B-ALL cells a survival advantage (Airoldi et al., 2006).

In multiple myeloma, SOCS1 expression is suppressed through promoter hypermethylation, which can be reversed through the activity of miR29b (Amodio et al., 2013).

In chronic lymphocytic leukemia (CLL), NF-κB signaling is regulated by multiple miRNAs, including miR-9-3a and miR708, both of which are downregulated through DNA hypermethylation (Wang L. Q. et al. 2013; Baer et al., 2015). This leads to aberrant NF-κB signaling.

#### Histone Methylation

Rearrangement of mixed-lineage leukemia genes in acute myeloid leukemia (AML) leads to aggressive hematopoietic malignancies. The IL-3Rα expression is dependent on Jmjd2/Kdm4 through a mechanism involving H3K9me3 removal from the gene promoter (Agger et al., 2016). It has also been shown that methyltransferase SUV39H1 is directly involved in resistance to TGF-β signaling in AML. Deregulation of TGF-β direct targets p21 and p15 through SUV39H1-mediated H3K9me3 marks leads to inhibition of cell cycle arrest and gives leukemic cells a proliferative advantage (Ruscetti et al., 2005). Furthermore, SETDB1 promotes heterochromatin formation and immune evasion in AML. Knockdown of SETDB1 leads to induction of the interferon immune response, thus qualifying SETDB1 as a fundamental leukemic survival protein (Schultz et al., 2002; Monaghan et al., 2019).

### Breast Cancer

Breast cancer is one of the common cancers reported worldwide. Clinically, breast cancers are categorized into three basic therapeutic groups, which include: estrogen receptor (ER) positive group, which is the most diverse and numerous, the HER2 amplified group with effective therapeutic targeting of Her2, and the triple-negative breast cancer with only chemotherapy options and have a high incidence of BRAC1 mutation in the patients.

#### DNA Methylation

The interaction between the chemokine CXCR4 and its ligand, CXCL12, is linked to cell proliferation, survival, invasion, and metastasis in various forms of cancer, such as breast cancer, where the upregulation of CXCR4 in cancer tissue is also accompanied by peak expression of its ligand CXCX12, at sites of common metastasis (Müller et al., 2001). CXCR4 is known to be hypomethylated and overexpressed, while CXCL12 is hypermethylated and absent in breast cancer cell lines and primary tumors themselves. Patients with both CXCL12 hypermethylation and CXCR4 hypomethylation showed shorter overall survival and disease-free survival (Ramos et al., 2011).

Interestingly, chemotherapy in breast cancer patients was shown to be associated with significantly decreased methylation at eight CpG sites in peripheral blood mononuclear cells and increased levels of TNFR2 and IL-6 (Smith et al., 2014). Inflammation and fatigue are known to be common effects of chemotherapy.

#### Histone Methylation

One of the first identified demethylases, LSD1, facilitates demethylation of H3K4/K9 and is associated with nuclear receptors. H3K4 demethylation by LSD1 was previously shown to inhibit inflammation (Shi et al., 2004, p. 1). CoREST1 on coordination with LSD1 promotes expression of VEGF-A and proinflammatory factors CCL2/MCP-I and CXCL16 and contributes to angiogenesis and tumor inflammatory responses in breast cancer (Zhao and Shilatifard, 2019). A recent study showed that G9a promotes breast cancer recurrence by inhibiting pro-inflammatory signaling pathway. G9a activity is essential to downplay the expression of pro-inflammatory cytokines, including TNF, by H3K9 methylation in the gene promoters. G9a repression triggers re-expression of the pro-inflammatory cytokines leading to activation of p53 and necroptosis. The study showed that receptor interacting protein kinase-3 (RIPK3) expression is upregulated in recurrent tumors, which makes it sensitive to necroptosis following G9a suppression in breast cancer. Histone methyl-modifying enzymes are now being considered as potential therapeutic targets against cancer, by inhibiting the inflammatory response (Mabe et al., 2020).

### Cancers of the Reproductive Systems

Of the cancers of the reproductive systems, the most common are endometrial, ovarian, and cervical cancers in females and prostate and testicular cancers in males. While less is known about the link between inflammation and epigenetics in these cancers, there is growing evidence to show the presence of this link and the similarities with other cancers. In particular, cervical cancer in females, which is often linked to infection by the human papillomavirus (HPV), which indicates the intricate relationship between infection, inflammation, and cancer; in cervical cancer, anti-viral inflammation is required to prevent cancer development, but it is also necessary to limit the effects of inflammation on cervical tissue (Cohen et al., 2019).

#### DNA Methylation

In cervical cancer tissues, methylation of the IFN-γ gene is significantly higher than in healthy cervical tissue, resulting in reduced expression (Ma et al., 2020). The viral oncoprotein E6/E7, produced by human papillomavirus 16 (HPV16), can also induce DNMT activation, DNA methylation, and downregulation of IFN-κ, which is also downregulated in cervical cancer biopsies compared to healthy tissue (Rincon-Orozco et al., 2009; Alfaro-Mora et al., 2019). In ovarian cancer, many genes, including the pro-inflammatory TNF and CXCR4, are hypomethylated and overexpressed (Gong et al., 2020).

#### Histone Methylation

Previous studies show that in prostate cancers, TGF-β promotes H3K4me3 and Retinoblastoma binding protein 5 (RbBP5) recruitment to the Snail promoter by association with Smad2/3 and CBP, leading to enhanced Snail expression in the cancer cells (Li et al., 2016).

### Other Cancers

In esophageal squamous cell carcinoma (ESCC), differentially methylated CpG sites between cancer tissue and healthy tissue were found in genes in the IL10 signaling pathway; IL-6 was found to be hypomethylated, while IL-1α was found to be hypermethylated (Lima et al., 2011). TGFβRII is likewise downregulated through promoter hypermethylation in ESCC, where it can regulate proliferation of ESCC cell line by G2/M arrest; treatment with 5-aza was able to restore its expression (Ma et al., 2020).

CXC chemokine receptor 4 is also hypermethylated and transcriptionally repressed in melanoma, as is CCR7; treatment with 5-Aza increases the expression of both these chemokines. In line with this, 5-Aza treated cells also showed increased migration in response to treatment with CCL21 and CXCL12, the ligands for CCR7 and CXCR4, respectively, indicating a functional rescue upon demethylation (Mori et al., 2005).

## Targeting Inflammation in Cancer

### DNA Methylation: Targeting Options and Therapeutics

The vast majority of genes that are deregulated through aberrant DNA methylation, including those that are involved in inflammatory signaling, tend to be hypermethylated. Consequently, inhibitors of DNA methylation are well developed and the development of DNMT inhibitors for the treatment of cancer has also made some progress. There are multiple generations and iterations of DNMT inhibitors that are now being developed or are in use. This includes Azacitidine, Decitabine, Zebularine, (-)-epigallocatechin-3-gallate, MG98, RG108, and Procainamide (Brueckner et al., 2007; Gravina et al., 2010; Gnyszka et al., 2013). However, the full extent of the effects of many of these drugs is not known, and problems include serious side effects, the development of resistance, and partial or no response in a group of patients. There also tends to be a change in global CpG methylation patterns as the result of using DNA inhibitors, which may pose a problem if new sets of genes are deregulated (Giri and Aittokallio, 2019). Another point to consider is that, although DNA hypermethylation underlies the deregulation of most inflammatory signaling genes, there are some that are hypomethylated rather than hypermethylated. The use of DNA inhibitors is, therefore, limited.

Nevertheless, better understanding of the mechanism of action and global effects of these inhibitors will allow us to implement more suitable dosing regimens in combination with other drugs.

### Histone Methylation: Targeting Options and Therapeutics

Histone methylation can contribute as potential therapeutic targets in digestive cancers. Inhibition of EZH2-mediated H3K27me and G9a-mediated H3K9me/H3K are being considered as important strategic targets. GSK343, the drug that suppresses EZH2 activity and reduces H3K27me3 expression was shown to potentially recover intestinal inflammation and also delayed onset of colitis-associated cancer (Zhou J. et al., 2019). DZNep (3-deazenplanocin A) which is a chemical inhibitor of S-adenosylhomocysteine hydrolase, suppresses histone methyltransferases including EZH2, and leads to marked reduction in cell proliferation and migration in colorectal cancer (Cheng et al., 2012). DZNep can also alter miR-663a and miR-4787-5p expression in turn suppressing TGFb1-induced EMT signaling in pancreatic cancers (Mody et al., 2016). EZH2 inhibitor, GSK126 can promote infiltration of functional CD8 T-cells by epigenetic reprogramming and significantly decrease HCC growth (Wei et al., 2019). GSK126 increases the number of myeloid-derived suppressor cells (MDSCs) and decreases CD4+ and IFNΥ CD8+ T-cells, which is associated with antitumor immunity in colorectal cancer, via EZH2-mediated H3k27me3 levels (Huang et al., 2019).

In the context of EMT, TGF-b1 treatment in gastric cancer cells promotes the expression of JARID1A demethylase, which is recruited by p-SMAD3 to CDH1 promoter, leading to gene silencing and promoting malignancy (Liang et al., 2015). EZH2 methyltransferase inhibitors that are now in clinical trials can be used with extracellular signal-regulated kinase (Erk) inhibitors to suppress TGF-β induced EMT (Olea-Flores et al., 2019).

## The Crosstalk: Inflammation-Induced Changes in Methylation

It is important to recognize that there also exists a crosstalk between inflammation and altered epigenetics. While altered epigenetic function may be the cause of altered expression and function of many inflammatory signaling networks, there is equal evidence, if not more, to show that the opposite is also true: inflammation and inflammatory signaling can cause aberrant methylation. It is crucial to recognize that targeting epigenetic regulators using small molecule modulators may not always be useful in targeting inflammatory networks; sometimes these lie upstream of the methylation aberrations and may have other underlying causes that may need to be identified for treatment.

Perhaps the best-known type of cancer linked to chronic inflammation is *Helicobacter pylori-*linked gastric cancer. *H. pylori* inject cytotoxin-associated antigen A (CagA) into host cells and cause inflammatory stress within gastric mucosa through activation of pathways including NK-κB, activator protein-1 (AP1), phosphoinositide 3-kinase (PI3K), STAT3, Wnt/beta-catenin, and COX-2. Inflammation-induced DMNT upregulation is thought to lead to the deregulation of many downstream targets. Studies have demonstrated the presence of aberrant methylation at the promoter of multiple genes in the gastric mucosa cells during *H. pylori* infection, including methylation-induced production of pro-inflammatory genes such as NOS2, IL1B, and TNF (Maeda et al., 2017). Compared to gastric mucosa upon *H. pylori* eradication, as well as compared to non-cancerous gastric tissue, there is hypermethylation at many of these loci. In a gerbil model, these changes are thought to be linked, not to the infection itself, but to the infection-induced inflammatory response, as methylation changes temporally reflected the expression levels of inflammation-related genes such as CXCL2, IL-1β, NOS2, and TNFα, and treatment with an anti-inflammatory drug (cyclosporin A) led to a blockade in the DNA methylation pattern changes previously seen without affecting colonization by *H. pylori* (Kurkjian et al., 2008; Niwa et al., 2010). The changes in DNA methylation seen in gastric epithelial cells were induced by bacteria-induced macrophage-produced NO, once again showing the link between inflammatory signaling and the induction of aberrant DNA methylation (Katayama et al., 2009, p. 3). In gastric cancer, *H. pylori* and Epstein-Barr virus (EBV)-induced chronic inflammation is known to induce aberrant methylation in the gastric mucosa, resulting in gene profile changes that promote tumorigenesis, including changes in the expression of target genes such as *p16INK4A, LOX, CDH1, IL-1β, IL-8, NOS2*, and *TNF* (Matsusaka et al., 2014).

In inflammatory bowel disease and colitis-associated CRC, which is characterized by chronic inflammation, there is an altered methylation profile when compared to sporadic CRC. In colitis-associated cancer, NF-κB-induced release of TNF and IL6 from immune cells is thought to induce NF-κB and STAT3-dependent altered signaling in epithelial cells of the gastric mucosa, including increased DNMT expression and changes to the methylome of the epithelial cells (Hartnett and Egan, 2012). DNMT expression was found to be higher than in sporadic CRC, and this increase was found to be linked to IL6 signaling. In addition, IL6-induced upregulation of DMNT1 has been linked to the hypermethylation and downregulation of SOCS3 (Li et al., 2012). IL6-induced upregulation of DNMT1 was also found to result in hypermethylation and silencing of genes linked to tumor suppression, adhesion, and apoptosis, including PAI-1, IL-4, Maspin, and IRF-7 (Foran et al., 2010). IL6 has also been shown to induce the cytochrome P450 gene CYP1B1 through DNA methylation-dependent suppression of microRNA miR27b (Patel et al., 2014). Hence, it is thought that inflammation induces a novel epigenetic profile, which thereby affects progression of CRC (Foran et al., 2010; Abu-Remaileh et al., 2015; Pekow et al., 2019). Altered methylation profile as the result of chronic inflammation is likewise the case in HCC (Stoyanov et al., 2015), where crosstalk between inflammation and epigenetic mechanisms can result in a positive feedback loop that advances inflammation and tumor progression (Sceusi et al., 2011; Martin and Herceg, 2012).

Interestingly, while some studies have reported the downregulation of COX-2 by promoter methylation in HCC, others have shown, using a transgenic COX-2 mouse model, that COX-2 is sufficient to induce HCC in a number of ways, including through the induction of hypermethylation and downregulation of TET1. TET1 converts 5m to 5-hydroxymethylcytosine (5hmC), so downregulation of TET1 results in hypermethylation and repression of several tumor suppressor genes (Chen et al., 2017).

In the IL-6-responsive human multiple myeloma cell line KAS 6/1, IL6 maintains promoter methylation of the tumor suppressor gene p53 through an induction of DMNT1 (Hodge et al., 2005). In addition, the circulating levels of IL6 appear to be associated with the methylation of several candidate genes, including MGMT, RARβ, RASSF1A, CDH13, SOCS1, USP2, TMEM49, SMAD3, DTNB, and IL-6 itself, across tumor specimens and peripheral blood samples. Furthermore, histone methyltransferase EZH2 is induced by IL-6 in IL-6 dependent MM cell lines (Croonquist and Van Ness, 2005).

Higher levels of methylation of EEF2 and SOCS1 were associated with higher levels of TNFα [reviewed by Gonzalez-Jaramillo et al. (2019)]. In AML, it has been suggested that stem-cell-like epitypes that lack a dominant driver mutation may be making use of pro-inflammatory signaling for AML cell survival and proliferation and that DNA methylation clustering can be used to identify a subset that makes use of the STAT inflammatory pathway (Giacopelli et al., 2021).

In triple-negative breast cancer, acetylated STAT3 associates with DMNT1 and promotes aberrant promoter hypermethylation (Lee et al., 2012). In basal-like breast cancer, NF-κB drives the repression of the Ten-eleven translocation enzymes TET1, which induces DNA demethylation by converting 5mC to 5hmC. This is also associated with activation of immune pathways and with tumor infiltration by immune cells. This link between NF-κB, immune cell infiltration, and TET1 suppression, leading to global changes in the methylome, is also found in melanoma, lung cancer, and thyroid cancer (Collignon et al., 2018).

In ovarian cancer cells, TGF-β-induced methylation of CpG islands located in or near promoters of genes involved in EMT and cancer progression was achieved through TGF-β-dependent upregulation of DNMTs (Cardenas et al., 2014). Interestingly, TGF-β can also inhibit DNMT1 and DNMT3a expression and global DNMT activity. In a rat model, it was shown to upregulate Collagen type I alpha I (COL1A1) by this mechanism (Pan et al., 2013). In cervical cancer, NO-dependent inflammation is known to deregulate DNA methylation of many target genes, including PTPRR (Su et al., 2017). Other hypermethylated targets include cell adhesion molecule 1 (CADM1) gene and T lymphocyte maturation associated protein (MAL) (Holubekova et al., 2020).

Changes in methylation patterns can be brought about by sex steroid hormones, treatment with which was shown to alter methylation patterns and expression of a variety of inflammatory signaling molecules in prostate cancer. Hypomethylated genes included CXCL12, CXCL5, CCL25, IL1F8, IL13RAI, STAT5A, CXCR4, and TLR5; while hypermethylated genes included ELA2, TOLLIP, LAG3, CD276, and MALT1 (Wang S. et al., 2016).

In oral squamous cell carcinoma (OSCC), IL6-induced inflammation alters global LINE1 hypomethylation, while also resulting in the hypermethylation of tumor suppressor genes such as CHFR, GATA5, and PAX6. Thus, inflammation-induced alterations to DNA methylation is thought to have important implications in tumor progression (Gasche et al., 2011).

IL1β is known to be highly expressed in the tumor microenvironment of various cancer types, and drives many malignant processes such as initiation, proliferation, and metastasis. IL1 is also key for angiogenesis and tumor growth and has been linked to metastases in various forms of cancer (Elaraj et al., 2006; Voronov et al., 2007). Similarly, IL8 is also known to be constitutively produced by cancer cells and cell lines and plays a role in tumor growth and metastasis (Xie, 2001). IL6 is a key pro-inflammatory cytokine that drives chronic inflammation in a number of ways and has been linked to poor survival outcomes in various forms of cancer (De Vita et al., 1998). Similarly, in a mouse model of gastric cancer, IL1β signaling was linked to promoter methylation and transcriptional repression of E-cadherin, a gene that is critical in preventing cell migration and metastasis (Huang et al., 2016). NF-κB promotes the expression of programmed death ligand 1 (PD-L1) through demethylation of its promoter. PD-L1 mediates a negative feedback of lymphocyte activation, a loop that is exploited by tumors for immune evasion and survival. The upregulation of PD-L1 is also linked to EMT (Antonangeli et al., 2020, p. 1). An EWAS-identified locus in the NLR Family CARD Domain Containing 5 (*NLRC5)* gene was associated with CXCL11, CXCL9, IL-12, and IL-18 levels (Ahsan et al., 2017). NLRC5 is involved in interferon-linked innate immunity through its ability to regulate MHC Class I receptors.

## Conclusion

The crosstalk between inflammation and epigenetic rewiring is one that is coming to be increasingly understood as posing unique challenges and opportunities for therapeutic development in cancer. Epigenetic rewiring by cancer cells allows them to manipulate and exploit several processes and phenomena that give them a survival advantage. Epigenetic modulators constitute a singularly useful class of enzymes for cancer cells, given their potentially wide range of targets and their reversible effects. A single modulator can have a host of target genes involved in a whole host of processes, so that alteration of that modulator alone can be used to achieve a wide range of downstream effects. This property of epigenetic modulators, however, also makes them useful targets for development of therapeutics. Inflammatory signaling networks are often deregulated in cancer through epigenetic means, meaning that the modulators can be targeted to treat inflammation, along with other cancer phenotypes regulated by that modulator. However, as discussed above, the opposite also happens; epigenetic rewiring may be a result, rather than the cause, of inflammatory dysregulation, so that it may be necessary to develop other means to target this oncogenic phenotype. It is also necessary to consider the global effects of epigenetic drugs. It has been shown that the use of DNMT inhibitors, for example, causes altered global gene profiles, which may result in the undesired activation or suppression of an entirely new set of genes (Giri and Aittokallio, 2019). Understanding the overall effects of these drugs requires further investigations.

Many cancers make use of similar alterations to give themselves a survival advantage, and it is therefore unsurprising that certain factors are overexpressed or underexpressed across an array of different cancers through similar mechanisms. For example, BRAK/CXCL14 is a chemokine that is constitutively produced by most tissue types but has been found to be depleted in a variety of human cancers and tumor cell lines. As a potent inhibitor of angiogenesis as well as a powerful chemoattractant for monocytes and dendritic cells, it is downregulated by cancers to allow the critical processes of angiogenesis and tumor infiltration by immune cells (Shellenberger et al., 2004; Shurin et al., 2005). The downregulation of CXCL14 is achieved, at least in part, through the hypermethylation of CpG island sequences in the CXCL14 gene promoter. Consequently, treatment using 5-Aza results in increased CXCL14 production and chemoattractant activity of conditioned medium (Song et al., 2010, p. 14). Similarities between various forms of cancer allow us to take the lessons and therapeutics developed from one form and attempt to apply it to others.

However, cytokines and chemokines have pleiotropic roles and may be exploited in different ways by different cancers to achieve survival, growth, and metastasis. For example, the promoter of IL10 has been shown to be either hypermethylated or hypomethylated depending on the type of cancer. A study that examined the methylation status of the IL10 family of genes across colon, kidney, lung, stomach, and breast cancer datasets found that these genes are typically hypomethylated (Shen et al., 2012), but this is not always the case. This underlines to need to exercise caution when trying to apply the principles and strategies used for one form of cancer to deal with another.

Even within a single type of cancer, there may be subsets of populations that show differential expression of certain genes. For example, while COX-2 is generally upregulated in CRCs, there is a small population in which it is downregulated through promoter hypermethylation, and this downregulation, in fact, is linked to poorer survival outcomes (Toyota et al., 2000; Song et al., 2001; Kikuchi et al., 2002; Hur et al., 2003; Soumaoro et al., 2004; Ogino et al., 2008; Wang and Dubois, 2010; Fernández-Alvarez et al., 2012). Using DNMT inhibitors would, therefore, exacerbate this effect in this group of patients.

Another thing to consider is the fact that the mechanisms by which histone methylation regulates gene expression are still debated. Detailed mechanisms linking H3K4me3 to upregulation in gene expression are still to be explored. Crosstalk between histone arginine methylation and lysine methylation are important subjects to be explored in the contexts of cancer and inflammation. The in-depth mechanisms of these interactions and upstream events along with the recruitment of the histone modifiers should be looked in greater detail for development of potent epi-drugs. Furthermore, with global changes in epigenetic marks, it can be difficult to disentangle cause from effect. H3K4me3 may be the “cause” rather than “effect” of upregulated gene expression. Likewise, as previously mentioned, it has been suggested that the *de novo* methylation may simply be a part of genome-wide process in a distinct subgroup of cancers characterized by a profound methylator phenotype (Sato et al., 2005). In other words, it may not always be possible to recognize the functional effect of certain methylation signatures through genome-wide screens, and changes in methylation may not necessarily lead to biologically significant changes in gene expression or activity, but may simply be a result of other changes.

Finally, it is important to recognize the interplay between groups of enzymes that perform opposing functions. For example, both DNMTs and TETs have been implicated in cancer, as both global hypomethylation and regional hypermethylation have been identified (Ehrlich, 2002; Szyf, 2003). It is important to understand both the global as well as the gene-specific roles of these two antagonistic groups of enzymes before attempting to target one or the other for therapy. After all, hypomethylation can in theory be attributed to either reduced DNMT activity or increased TET activity, and the opposite holds true for hypermethylation.

## Future Perspectives

The latest technological advancements in whole-genome transcriptomics analysis and epigenomic profiling will be crucial in the development of targeted therapeutic strategies. Despite their shortcomings, methylation signatures may still have some prognostic value. DNA methylation is critical for lineage specificity and cell differentiation, particularly for hematopoiesis and for the development of the myeloid-derived suppressor cells that are generally produced in response to tumor secreted factors, and which are linked to cancer-associated inflammation. Immunomethylomics, or methylation profiling of the immune cells using DNA from archival peripheral blood may be developed as a potential prognostic tool for solid tumors as well as in lymphatic/hematopoietic cancers, in which differential methylation and differential variability in methylation are associated with tumor progression and outcomes (Kelsey and Wiencke, 2018; Domingo-Relloso et al., 2021). In addition, integrating expanded DNA methylation data with somatic mutation data and gene expression has allowed the identification of “triple-evidenced” genes, which are differentially expressed, differentially methylated, and associated with somatic mutation in different forms of cancer, which could be further investigated as prognostic markers or therapeutic targets (Fan et al., 2019). Large, representative datasets make the identification of targets much more robust and reliable, indicating the need for collaboration, sharing, and meta-analysis of datasets across populations that may differ in terms of age, race, sex, and indeed, various other factors. This will be needed in order to extract meaningful, reproducible data that are both widely applicable and specific to certain populations. For instance, it is known that some populations are more susceptible to chronic inflammation and certain types of cancers, and these factors must be taken into consideration (Ranjit et al., 2007; Stepanikova et al., 2017; Özdemir and Dotto, 2017; Schmeer and Tarrence, 2018; Meaney et al., 2019; Yeyeodu et al., 2019).

Much of what we understand about inflammation and inflammatory pathways in cancer comes from research on other diseases in which inflammation is the most defining characteristic. Many allergic, autoimmune, and age-related conditions are characterized by major aberrations in inflammatory networks that can inform research on inflammation in cancer as well. The use of drugs that are used to treat inflammation in other pathologies may well be used along with existing cancer therapeutics in order to take a more holistic treatment approach. For example, it has been shown in lung cancer that the combined treatment of aspirin, a commonly used anti-inflammatory drug, and radiotherapy, resulted in a synergistic reduction of cell viability, partly through downregulation of COX-2 (Sun et al., 2018). Drugs that target inflammatory enzymes, such as non-steroidal anti-inflammatory drugs (NSAIDs), which target COX2, could also have a more complex role to play if inflammation lies upstream of epigenetic changes and altered genome profiles. In addition to drugs, dietary anti-inflammatory natural compounds, such as Vitamin C, D, and E all have effects on both inflammation and epigenetics, particularly DNA methylation (Saccone et al., 2015; Castellano-Castillo et al., 2018; Gerecke et al., 2018; Zappe et al., 2018; Yang et al., 2019). However, further work will be required to understand which combinations of drugs work together.

## Author Contributions

DD and NK contributed equally to the writing of this article. All authors contributed to the article and approved the submitted version.

## Conflict of Interest

The authors declare that the research was conducted in the absence of any commercial or financial relationships that could be construed as a potential conflict of interest.

## Publisher’s Note

All claims expressed in this article are solely those of the authors and do not necessarily represent those of their affiliated organizations, or those of the publisher, the editors and the reviewers. Any product that may be evaluated in this article, or claim that may be made by its manufacturer, is not guaranteed or endorsed by the publisher.

## Abbreviations

5mC-5: methylcytosine
ADP: adenosine diphosphate
AIM2: absent in melanoma 2
ALK: anaplastic lymphoma kinase
ALK: anaplastic lymphoma kinase
AML: acute myeloid leukemia
ANXA2: annexin A2
AP1: activator protein 1
B-ALL: B-cell acute lymphoblastic leukemia
BMDC: bone marrow-derived dendritic cells
CADM1: cell adhesion molecule 1
CAF: cancer-associated fibroblast
CagA: cytotoxin-associated gene A
CBP: CREB-binding protein
CCL2: C-C motif chemokine ligand 2
CDH1: cadherin-1
CDH13: cadherin 13
CHFR: checkpoint with forkhead and ring finger domains
CoREST1: corepressor for RE1 silencing transcription factor/neural-restrictive silencing factor
COX: cyclooxygenase
CRC: colorectal cancer
CRP: C-reactive protein
CXCL: C-X -C motif chemokine ligand
CXCR: C-X -C motif chemokine receptor
DC: dendritic cell
DNMT: DNA methyltransferase
DTNB: dystrobrevin beta
DZNep: 3-deazaneplanocin A
EBV: Epstein–Barr virus
EEF2: eukaryotic elongation factor 2
EEF2: eukaryotic translation elongation factor 2
EGFR: epidermal growth factor receptor
EGFR: epidermal growth factor receptor
ep-CAM: epithelial cell adhesion molecule
Erk: extracellular signal regulated kinase
EWAS: epigenome wide association studies
Ezh2: enhancer of zeste 2
EZH2: enhancer of zeste 2 polycomb repressive complex 2 subunit
FOXM1: forkhead box protein M1
HBx: hepatitis B X protein
HCC: hepatocellular carcinoma
HPV: human papilloma virus
IFN: interferon
IL: interleukin
JAK: Janus kinase
JARID2: Jumonji, AT rich interactive domain 2
JMJD3: Jumonji domain-containing protein D3
KMT: lysine methyltransferase
KMT1B: lysine N-methyltransferase 1B
LAG3: lymphocyte-activation gene 3
LOX: lipoxygenase
LSD1: lysine-specific histone demethylase 1A
LTF: lactotransferrin
LY86: lymphocyte antigen 86
MAL: myelin and lymphocyte protein
MALT1: mucosa-associated lymphoid tissue lymphoma translocation protein 1
MCP-I: monocyte chemoattractant protein-1
MDSC: monocyte-derived suppressor cell
MEN1: multiple endocrine neoplasia type1
MGMT: O^6^-methylguanine DNA methyltransferase
MHC: major histocompatibility complex
MICA: MHC class I polypeptide–related sequence A
MMP-9: matrix metallopeptidase 9
NF-κB: nuclear factor kappa light chain enhancer of activated B cells
NK: natural killer
*NLRC5*: NOD-like receptor family CARD domain containing 5
NO: nitric oxide
NOS2: nitric oxide synthase
NSAID: nonsteroidal anti-inflammatory drug
NSCLC: non-small cell lung cancer
NTRK: neurotrophic tropomyosin receptor kinase
NTRK: neurotrophin receptor kinase
OSCC: oral squamous cell carcinoma
PAI-1: plasminogen activator inhibitor 1
PAX6: paired box 6
PcG: polycomb group
PD-L1: programmed death-ligand 1
PGE2: prostaglandin E2
PI3K: phosphatidylinositol 3-kinase
PPAR-γ: peroxisome proliferator-activated receptor gamma
PRC2: polycomb repressive complex 2
PTM: post translational modification
PTPRC: Protein Tyrosine Phosphatase Receptor Type C
PTPRR: Protein Tyrosine Phosphatase Receptor Type R
RARβ: retinoic acid receptor beta
RASSF1: Ras association domain-containing protein 1
RbBP5: RB binding protein 5, histone lysine methyltransferase complex subunit
RIPK3: receptor interacting serine/threonine kinase 3
S100A9: S100 calcium-binding protein A9
SAM: S-adenyl methionine
SETDB1: SET domain bifurcated histone lysine methyltransferase 1
Smad3: mothers against decapentaplegic homolog 3
SOCS1: suppressor of cytokine signaling 1
SRC: tyrosine-protein kinase
SSI: systematic inflammation
STAT3: signal transducer and activator of transcription 3
T reg: regulatory T cell
TAM: tumor associated macrophage
TET1: ten-eleven translocation methylcytosine dioxygenase 1
TGF: tumor growth factor
Th: T helper
TME: tumor microenvironment
TNFR2: tumor necrosis factor receptor 2
TNFs: tumor necrosis factors
TOLLIP: toll interacting protein
ULBP: UL16 binding protein 1
USP2: ubiquitin specific peptidase 2
VEGF: vascular endothelial growth factor
YAP1: yes-associated protein 1.
